# Association between severity of prenatally diagnosed hydronephrosis and receipt of surgical intervention postnatally among patients seen at a fetal-maternal center

**DOI:** 10.1186/s12894-021-00822-7

**Published:** 2021-04-07

**Authors:** Zoë G. Baker, Arthi Hannallah, Melissa Trabold, Danielle Estell, Cherry Deng, Andy Y. Chang, S. Scott Sparks, Roger De Filippo, Evalynn Vasquez

**Affiliations:** 1 Division of Urology, Children’s Hospital Los Angeles, Los Angeles, CA USA; 2 Keck School of Medicine of Medicine of USC, University of Southern California, Los Angeles, CA USA; 3 Thomas J. Long School of Pharmacy, University of the Pacific, Stockton, CA USA

**Keywords:** Hydronephrosis, Prenatal, Surgery, Fetal, Diagnosis

## Abstract

**Background:**

Hydronephrosis (HN) is the most common abnormality detected on prenatal ultrasound. This study sought to stratify outcomes of patients by severity of prenatal HN with postnatal outcomes.

**Methods:**

This was a retrospective review of patients referred to a tertiary care fetal-maternal clinic with diagnosis of prenatal HN from 2004 to 2019. HN severity was categorized as mild, moderate, or severe. Data were analyzed to determine the association between HN severity and surgical intervention. Decision for surgery was based on factors including history of multiple urinary tract infections, evidence of renal scarring, and/or reduced renal function. Surgery-free survival time was represented by the Kaplan–Meier method, and hazard ratios were calculated using the log-rank test.

**Results:**

131 kidneys among 101 infants were prenatally diagnosed with hydronephrosis; 35.9% had mild HN, 29.0% had moderate HN, and 35.1% had severe HN. 8.5% of patients with mild HN, 26.3% of patients with moderate HN, and 65.2% of patients with severe HN required surgery. Patients with severe HN were 12.2 (95% CI 6.1–24.4; *p* < 0.001) times more likely to undergo surgery for HN than patients with mild HN and 2.9 (95% CI 1.5–5.3; *p* = 0.003) times more likely to undergo surgery than patients with moderate HN. Patients with moderate HN were 4.3 times more likely to require surgery than patients with mild HN (95% CI 1.5–12.9; *p* = 0.01). Median age at surgery was 11.8 months among patients with mild HN (IQR 11.7–14.1 months), 6.6 months among patients with moderate HN (IQR 4.2–16.4 months), and 5.4 months among patients with severe HN (3.7–12.4 months).

**Conclusion:**

Among this cohort of referrals from a fetal-maternal clinic, severity of HN correlated with increased likelihood of surgical intervention. Continued assessment of patients with prenatal HN should be evaluated to best determine the role of the pediatric urologist in cases of prenatal HN.

## Background

Hydronephrosis (HN) is a descriptive term for dilation of one or both kidneys. It is the most common prenatal abnormality detected by ultrasound [1] and is diagnosed in one to five percent of pregnancies [2, 3]. Increased prenatal screening has enabled physicians to diagnose prenatal HN more readily. Renal ultrasonography (US) and voiding cystourethrogram (VCUG) are often performed postnatally for physicians to assess renal function and consider treatment plans.

The Society for Fetal Urology (SFU) has categorized the severity of HN into grades 1–4. This system takes into consideration the degree of pelvic dilation, presence of calyceal dilation, and thinning of parenchymal tissue in the kidneys. Despite advances in prenatal ultrasound screening for HN, there is still some ambiguity in criteria for diagnosing severity and appropriate management. There is debate about the cutoff values for the SFU grading system, though efforts have been made by various professional groups to consolidate findings into a more comprehensible management guide for practicing urologists [4]. Another highly contested matter is the threshold at which surgical intervention is needed. Gender, age, and the presence of urological comorbidities are factors that must also be considered during the course of treatment.

After HN is detected on prenatal ultrasound, prenatal counseling with pediatric urologists is encouraged in order to form appropriate management plans. Urologists may recommend newborns with prenatal HN to be put on antibiotic prophylaxis in order to reduce the risk of urinary tract infections (UTIs) [5]. This is usually followed by monitoring with ultrasounds to assess the progression and potential resolution of HN. While current literature indicates that a large proportion of cases are stabilized or resolved following conservative management [1, 3, 6], studies have reported differing findings regarding the proportion of patients who go on to require surgery. A recent review found that the rate of complete resolution of prenatally-detected HN in the absence of surgery may range from 33 to 70% [7]. Discrepancies in the rates of patients who require surgery may be largely due to differences in HN severity between populations [8–13]. Variation in the proportion of patients with prenatally diagnosed HN who require surgery after birth may result in difficulty in counseling pregnant women on their child’s expected care trajectory. In some patients with HN, surgery may be indicated to prevent or manage recurrent UTIs, kidney dysfunction, ureteropelvic junction obstruction (UPJO) and/or vesicoureteral reflux (VUR) [1, 6–8, 13]. Common surgical interventions for HN, depending on the etiology of hydronephrosis, include pyeloplasties for UPJO, ureteral reimplantations for VUR, and in some cases, nephrectomies for symptomatic, nonfunctional kidneys [7, 9, 13, 14].

The aim of this study is to assess the number and proportion of patients diagnosed with mild (SFU Grade 1 or 2), moderate (SFU Grade 3), and severe (SFU Grade 4) prenatal HN at a fetal-maternal center that progress to surgery, and to assess the correlation between severity of prenatal diagnosis and probability of postnatal surgical intervention. Time to surgery by prenatal severity score will also be assessed. The results of this study may assist providers in delivering more accurate prenatal counseling to parents following identification of HN, illustrate the importance of prenatal visits, and may also aid pediatric urologists in taking appropriate precautions while identifying the etiology among pediatric patients with HN.

## Methods

Electronic medical records were reviewed among patients who received prenatal HN diagnoses by neonatologists and referred to pediatric urologists at a fetal-maternal center in a large urban setting between 2004 and 2019. Information on prenatal urologic diagnoses and severity of hydronephrosis was recorded from mothers’ pediatric urology notes, radiology reports, and/or radiology images. Patients’ demographic, clinical, and surgical information were similarly recorded and linked to their mothers’ data.

Patients were included in the study if severity of HN was recorded at the prenatal visit, they attended at least one urology appointment postnatally, and they were at least 12 months old at the time of data analysis. Patients with prenatal anteroposterior (AP) diameters of less than 10 mm were excluded, as this was the cut-off for patients to receive pediatric urology referrals. Patients were also excluded if they did not survive to term or were less than 12 months old at the time of data analysis. Patients were followed until the date of their surgical intervention, until the date their HN resolved, or until the date of their last follow-up with a pediatric urologist, up until age 6 years. Prenatal images were reviewed and hydronephrosis severity was assessed by each patient’s pediatric urologist. HN severity was classified as mild (SFU 1–2), moderate (SFU 3), or severe (SFU 4). Classifications were based on the status of the calices, extent of pelvic dilation, and presence of parenchymal atrophy. SFU grades of 1 and 2 were combined as some prenatal notes did not differentiate between the two and instead indicated “SFU grade 1/2.” Data on both kidneys were recorded among patients prenatally diagnosed with bilateral HN.

The probability of undergoing surgical intervention for HN was stratified by prenatally diagnosed HN severity. Patients’ diagnoses were classified postnatally. If a specific diagnosis was not given due to the transient nature of the patient’s HN, it was classified as transient. Decision for surgical intervention was based on the clinical characteristics of the patient including history of multiple recurrent urinary tract infections, evidence of renal scarring, and/or decreased renal function. Surgical interventions of interest included: pyeloplasty, nephrectomy, ureteral reimplantation, ureterostomy, ureterocele incision, and other urologic surgeries related to a diagnosis of HN. Among patients who underwent surgery, age at time of surgery was recorded. Kaplan–Meier curves and estimates with confidence intervals were created to assess the probability of receiving surgery versus proceeding with conservative management over time, stratified by HN severity. Whether patients were discharged from the clinic after resolution of HN without surgery or were receiving continued follow-up and monitoring for HN at the time of data analysis was also noted. Patients who did not undergo surgery and who had persistent HN at the time of data analysis were censored at the time of last clinic follow-up visit. Hazard ratios (HR) were calculated using the log-rank test, to determine differences in probability of surgery between patients with severe versus mild HN, severe versus moderate HN, and moderate versus mild HN. Etiology of HN by prenatal severity was also reported. Pearson X^2^ tests were utilized to evaluate whether there were any differences in probability of receiving surgery by patient gender, race/ethnicity, primary language, medical insurance type (public versus private), or vesicoureteral reflux (VUR) presence.

This study was reviewed and approved by the institutional review board at Children’s Hospital Los Angeles.

## Results

### Overall results

This study included 101 infants and 131 kidneys prenatally diagnosed with HN (Table 1). Mean gestational age at first detection of hydronephrosis was 23.5 weeks (IQR 20–31), though mothers were not referred to pediatric urology until AP diameter was at least 10 mm. There were no significant differences in gestational age at HN detection by HN severity. Postnatally, patients were followed for a median of 1.2 years (IQR 0.8–2.3 years). The majority of patients were male (n = 75, 74.3%), and 40% (n = 41) identified as Hispanic. Approximately 70% of patients were diagnosed with unilateral HN (n = 71), and 30% with bilateral HN (n = 30). Overall, 33.6% (n = 44) of hydronephrotic kidneys required surgical intervention, including 8.5% of kidneys diagnosed with mild HN, 26.3% of those diagnosed with moderate HN, and 65.2% of those diagnosed with severe HN (Table 2). Of the remaining kidneys, 30 (22.9%) spontaneously resolved, and 57 (43.5%) did not yet require surgical intervention and were being conservatively managed with serial ultrasound.

**Table 1:** Demographic and prenatal factors among patients prenatally diagnosed with hydronephrosis at fetal-maternal center and followed post-birth from 2004–2019 (n = 101)

Demographic/prenatal factor	n	%
Gender		
Male	75	74.3
Female	26	25.7
Race/ethnicity		
Hispanic	41	40.6
Non-Hispanic White	19	18.8
Other	28	27.7
Unknown	13	12.9
Parent primary language		
English	64	63.4
Non-English	36	35.6
Unknown	1	1.0
Medical insurance type		
Public	68	67.3
Private	33	32.7
Hydronephrosis side		
Unilateral	71	70.3
Bilateral	30	29.7
Prenatally-diagnosed hydronephrosis severity (among n = 131 kidneys)		
Mild	47	35.9
Moderate	38	29.0
Severe	46	35.1

**Table 2:** Number and rate of kidneys requiring surgical intervention among 131 kidneys diagnosed prenatally with mild, moderate, and severe hydronephrosis

Hydronephrosis severity	Total (n)	Surgical intervention (n)	Surgical intervention (%)
Mild	47	4	8.5%
Moderate	38	10	26.3%
Severe	46	30	65.2%
Total	131	44	33.6%

**Fig. 1:**
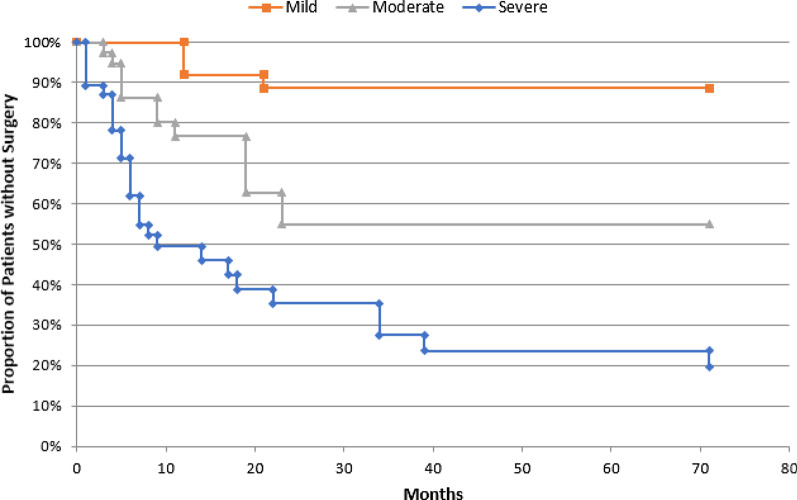
Kaplan–Meier curves indicating surgery-free survival time in months, by prenatally-diagnosed hydronephrosis severity type

The likelihood of requiring surgery varied significantly by prenatal HN severity. Additionally, among those patients who required surgery, age at surgery varied by HN severity; patients with moderate and severe HN generally underwent surgery at younger ages than patients with mild HN (Fig. 1). Patients with severe HN were 12.2 times more likely to require surgery than patients with mild HN (95% CI 6.1–24.4; *p* < 0.001), and 2.9 times more likely to require surgery than patients with moderate HN (95% CI 1.5–5.3; *p* = 0.003). Additionally, patients with moderate HN were 4.3 times more likely to require surgery than patients with mild HN (95% CI 1.5–12.9; *p* = 0.01). There was no significant difference in probability of receiving surgery by patient gender, race/ethnicity, primary language, or medical insurance type. Additionally, there was no significant difference in the probability of surgical intervention among patients with evidence of VUR, compared to those with no evidence of VUR (45.5% vs. 43.8%; X^2^ *p* = 0.72).

### Patients with mild prenatally diagnosed hydronephrosis

**Fig. 2:**
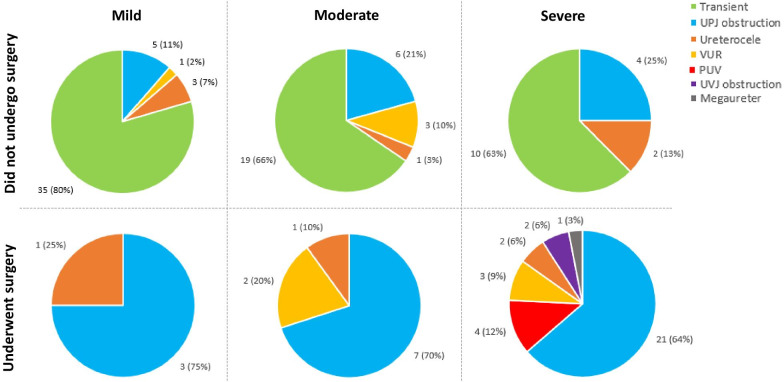
Number and percentage of hydronephrosis-related diagnoses among 101 patients, by prenatally-diagnosed hydronephrosis severity and receipt of surgical intervention

Among the 131 kidneys included in the study, 47 (35.9%) were prenatally diagnosed with mild HN. Most patients (74.5%, n = 35) who were diagnosed with mild HN had transient etiologies that self-resolved (Table 3; Fig. 2). Overall, four patients (8.5%) with mild HN required surgery for HN postnatally, which occurred at a median age of 11.8 months (IQR 11.7–14.1 months; Fig. 1). At the time of analysis, 20 kidneys with mild HN had spontaneously resolved, and 23 were still being followed with serial ultrasound. The Kaplan–Meier estimate indicates that 8.1% (95% CI 0.0–16.9%) of patients with mild HN require surgery by one year of age, and 11.4% (95% CI 0.8–22.0%) require surgery by six years of age (Fig. 1). HN-related diagnoses among patients who required surgery included UPJO in three cases, and ureterocele in one case (Table 3; Fig. 2). Three of these patients underwent pyeloplasty, and one patient underwent ureteral reimplantation, ureterocele incision, and bladder neck reconstruction (Table 3). In addition to transient etiologies, other etiologies that did not require surgical intervention among patients with mild HN included partial UPJO that did not meet the requirements for surgical intervention in five kidneys, VUR in three patients, and ureterocele in one patient.

**Table 3:** HN-related diagnoses, number of patients who required surgery, and surgical interventions among patients with mild, moderate, and severe prenatally-diagnosed HN

HN severity	HN-related diagnoses*	Total kidneys n	Kidneys requiring surgery n	Surgical intervention	
				Surgery type	n
Mild	UPJ obstruction	8	3	Pyeloplasty	3
	Ureterocele	2	1	Ureterocele incision, ureteral reimplantation, bladder neck reconstruction	1
	VUR	3	0	–	–
	Transient	35	–	–	–
	Total	47	4	–	–
Moderate	UPJ obstruction	13	7	Pyeloplasty	7
				Ureteroureterostomy	1
	VUR	5	2	Ureteral reimplantation	2
	Ureterocele	2	1	Nephroureterectomy	1
	Transient	19	–	–	–
	Total	38	10	–	–
Severe	UPJ obstruction	25	21	Pyeloplasty	17
				Partial nephrectomy	3
				Cutaneous ureterostomy	1
				Vesicostomy	1
	PUV	4	4	Valve ablation	4
	Ureterocele	4	2	Ureterocele incision	2
	VUR	3	3	Vesicostomy	2
				Ureteral reimplantation	1
	UVJ obstruction	2	2	Ureteral reimplantation	2
	Megaureter	1	1	Circumcision	1
	Transient	10	–	–	–
	Total	46	30	–	–

*Patients may have more than 1 diagnosis and may have undergone more than 1 type of surgical intervention

### Patients with moderate prenatally diagnosed hydronephrosis

A total of 38 kidneys (29.0%) were diagnosed with moderate prenatal HN, of which 10 (26.3%) required surgery (Table 2). Four kidneys with moderate HN spontaneously resolved, and the majority (n = 24) were still being followed for moderate HN at the time of data analysis. The Kaplan–Meier estimate indicates that 23.4% (95% CI 10.2–36.6%) of patients with moderate HN require surgery by one year of age, and 45.4% (95% CI 22.5–68.3%) may require surgery by six years of age (Fig. 1). Patients with moderate HN who required surgery were a median of 6.6 months old at the time of surgery (IQR 4.2–16.4 months). The majority of patients who required surgery had UPJO (n = 7; 70% of patients who underwent surgery; Table 3; Fig. 2). Additionally, one patient who required surgery had a ureterocele, and two had VUR. Most patients (n = 7) who required surgery underwent pyeloplasty, two patients underwent ureteral reimplantations, and one patient each underwent a ureteroureterostomy and a nephroureterectomy (Table 3). Patients who did not require surgery most frequently had a transient etiology of their HN (n = 19; Table 3; Fig. 2). Additionally, among patients who did not require surgical intervention, six had UPJO not meeting the requirements for surgical intervention, three had VUR, and one had a ureterocele.

### Patients with severe prenatally diagnosed hydronephrosis

Kidneys with severe HN accounted for 35.1% of kidneys included in the study (n = 46). Postnatal surgical intervention was required among 65.2% (n = 30) of kidneys with severe HN, and patients received surgery at a median of 5.4 months of age (3.7–12.4 months; Fig. 1). Of the remaining kidneys, six self-resolved and 10 were being followed conservatively. The Kaplan–Meier estimate indicates that 50.5% (95% CI 36.3–64.8%) of patients with severe HN require surgery by one year of age, and 80.3% (95% CI 66.0–94.7%) may require surgery by six years of age (Fig. 1). UPJO remained the most common HN-related diagnosis among patients with severe HN who underwent surgery, occurring in 70.0% (n = 21) of these patients (Table 3; Fig. 2). Other HN-related diagnoses among patients who underwent surgery included posterior urethral valves (PUV), ureterocele, ureterovesical junction obstruction (UVJO), VUR, and megaureter. Among the 30 kidneys requiring surgery, 17 underwent pyeloplasty (Table 3). Additional surgical interventions included valve ablation, partial nephrectomy, cutaneous ureterostomy, vesicostomy, ureterocele incision, ureteral reimplantation, and circumcision for recurrent urinary tract infections (Table 3). Among the 16 patients with severe prenatal HN who did not require surgical intervention, diagnoses included transient etiologies (n = 10), UPJO not meeting the requirements for surgical intervention (n = 4), and ureterocele (n = 2).

## Discussion

Prenatal HN is the most common prenatal abnormality detected on ultrasound and is a common referral to pediatric urologists [1]. How this HN can be managed depends on the severity of HN and concurrent findings that are identified in postnatal evaluation, generally by VCUG and renal US [4, 6]. The aim of this study was to stratify the proportion of patients with mild, moderate, and severe HN who eventually require surgical intervention in order to gain greater insight into how to adequately counsel families prenatally on the management of HN. There was a relatively homogenous spread of the severity of HN in this cohort of 101 patients (35.9% mild, 29.0% moderate, and 35.1% severe). It has been previously reported that 35–50% of patients with antenatal HN will have no HN on postnatal renal US [6]. The etiology of HN in our cohort also similarly reflected findings from previous studies. The most common pathological finding in our cohort was also UPJO, but the most common cause overall of HN was transient HN [6].

### Patients with mild prenatally diagnosed hydronephrosis

Among patients with mild prenatal HN, 74.5% had a transient etiology that resolved postpartum, none of whom had surgical intervention. As with previously reported studies, patients with transient HN determined to no longer be present postnatally may not warrant close urologic follow-up after a negative ultrasound result [4]. However, 8.5% of patients with mild prenatal HN did require surgical intervention, suggesting that postnatal follow-up of patients diagnosed with mild prenatal HN is warranted.

### Patients with moderate prenatally diagnosed hydronephrosis

As with the patients with mild HN, those with moderate HN who were determined to have transient HN did not require surgical intervention. While transient HN was less common in patients with moderate versus mild prenatal HN, it still occurred in half of all patients diagnosed with moderate prenatal HN. Approximately one quarter of all kidneys prenatally diagnosed with moderate HN required surgery. Time to surgery in this cohort was notable. The median time to surgery for patients with moderate HN was 6.6 months, compared to 11.8 months in those with mild HN and 5.4 months in those with severe HN. Liu et al. noted that the rate of pathology in those with moderate prenatal HN was 45.1% (25.3–66.6) [15]. This raises the question of why those with moderate HN were likely to undergo intervention around the same as those with more severe findings. While fewer patients with moderate HN underwent surgery overall compared to those with severe HN, one must consider what prompts practitioners to consider intervention particularly in this moderate group.

### Patients with severe prenatally diagnosed hydronephrosis

Like those in the mild and moderate cohorts, those patients with severe HN who were determined to have transient HN did not undergo surgical intervention. As one would expect, the patients with severe prenatal HN were significantly more likely to require some form of surgical intervention, which occurred at the earliest median time, as compared to mild and moderate cohorts. Among all kidneys with severe prenatal HN, almost 50% required surgical intervention due to UPJO. Surgical interventions required for HN-related diagnoses were also broader than patients with mild or moderate HN, including valve ablation, vesicostomy, cutaneous ureterostomy, partial nephrectomy, and circumcision for recurrent urinary tract infection in some cases. Data from this study suggest that patients with severe prenatal HN require close postnatal follow-up and may require surgery within the first six months of life.

### Limitations

This study is not without its limitations. Due to the retrospective nature of this study, classifications of HN severity were not standardized; while some providers used SFU grades, others used descriptions of “mild,” “moderate,” or “severe” to diagnose prenatal HN severity. Additionally, with only 101 patients in the cohort, our study is underpowered to be able to adequately stratify the severity of HN with the type of surgical procedure; however, it was possible to assess the association between increased HN severity and increased risk of surgical intervention of any type. The decision to recommend surgery was based on the patients’ clinical characteristics and the pediatric urologists’ judgements, which may vary depending on the surgeon. Additionally, as the median length of follow-up time in this study was 1.2 years, the Kaplan–Meier estimator for surgery-free survival through six years may be unreliable as it relies on data from few participants. The broad confidence intervals provided reflect the uncertainty of the six-year Kaplan–Meier estimates. For this reason, the six-year surgery-free survival estimate should be considered with caution, and the Kaplan–Meier estimator for surgery-free survival through one year is more reliable. Furthermore, assessment of outcomes among patients with prenatal HN in a multi-institutional setting would hold more impact in being able to determine how to manage the equivocal cases of prenatal HN.

## Conclusions

The experience from this maternal fetal health center provides further reassurance that patients with transient HN will not likely require surgical intervention in the future, despite severity. However, regardless of the nature of HN, those with moderate and severe HN do require further urologic work up beyond postnatal renal US to evaluate for potential pathologies and prevent infection or loss of renal function. Additionally, it reiterates the importance of prenatal counseling for close postnatal follow-up no matter the degree of prenatal hydronephrosis as it is difficult to predict the likelihood of surgery or resolution of HN on prenatal ultrasound alone. Even the mild form of prenatal HN may have pathology that requires surgical intervention. However, patients who are diagnosed with prenatal HN are significantly more likely to require surgical intervention and at an early age if they are found to have severe HN. Close follow-up and adequate family counseling in those with more severe HN prenatally are warranted.

## Data Availability

In order to uphold participant confidentiality and adhere by the guidelines stipulated by our IRB, data will not be made publicly available.

## Abbreviations

AP: Anteroposterior
CI: Confidence Interval
HN: Hydronephrosis
HR: Hazard Ratio
IQR: Interquartile Range
PUV: Posterior Urethral Valves
SFU: Society for Fetal Urology
UPJO: Ureteropelvic Junction Obstruction
UVJO: Ureterovesical Junction Obstruction
US: Ultrasonography
UTI: Urinary Tract Infection
VCUG: Voiding Cystourethrogram
VUR: Vesicoureteral Reflux

## References

[1] Yalcinkaya F, Ozcakar ZB (2019). Management of antenatal hydronephrosis. Pediatr Nephrol.

[2] Lee RS, Cendron M, Kinnamon DD, Nguyen HT (2006). Antenatal hydronephrosis as a predictor of postnatal outcome: A meta-analysis. Pediatrics.

[3] Arnaud A, Hossini SL, de Lara ST, Dobremez E, Chateil J, Harper L (2019). Managing children with hydronephrosis: Common pitfall during ultrasound follow-up to remember. Arch Dis Child.

[4] Kohno M, Ogawa T, Kojima Y, Sakoda A, Johnin K, Sugita Y, Nakane A, Noguchi M, Moriya K, Hattori M, Hayashi Y, Kubota M (2020). Pediatric congenital hydronephrosis (ureteropelvic junction obstruction): Medical management guide. Int J Urol..

[5] Braga LH, Mijovic H, Farrokhyar F, Pemberton J, DeMaria J, Lorenzo AJ (2013). Antibiotic prophylaxis for urinary tract infections in antenatal hydronephrosis. Pediatrics.

[6] Woodward M, Frank D (2002). Postnatal management of antenatal hydronephrosis. BJU Int.

[7] Passoni NM, Peters CA (2020). Managing ureteropelvic junction obstruction in the young infant. Front Pediatr.

[8] Chertin B, Pollack A, Koulikov D, Rabinowitz R, Hain D, Hadas-Halpren I, Farkas A (2006). Conservative treatment of ureteropelvic junction obstruction in children with antenatal diagnosis of hydronephrosis: Lessons learned after 16 years of follow-up. Eur Urol.

[9] Sadeghi-Bojd S, Kajbafzadeh A, Ansari-Moghadam A, Rashidi S (2016). Postnatal evaluation and outcome of prenatal hydronephrosis. Iran J Pediatr.

[10] Yang Y, Hou Y, Niu ZB, Wang CL (2010). Long-term follow-up and management of prenatally detected, isolated hydronephrosis. J Pediatr Surg.

[11] Ulman I, Jayanthi VR, Koff SA (2000). The long-term followup of newborns with severe unilateral hydronephrosis initially treated nonoperatively. J Urol.

[12] Arena S, Chimenz R, Antonelli E, Peri FM, Romeo P (2018). A long-term follow-up in conservative management of unilateral ureteropelvic junction obstruction with poor drainage and good renal function. Eur J Pediatr.

[13] Leong SY, Afolabi TM, Tsu LV (2016). The management of antenatal hydronephrosis U.S. Pharmacist.

[14] Sung J, Skoog S (2012). Surgical management of vesicoureteral reflux in children. Pediatr Nephrol.

[15] Liu DB, Armstrong WR, Maizels M (2014). Hydronephrosis: prenatal and postnatal evaluation and management. Clin Perinatol.

